# Successful Prompt Diagnosis of Strongyloidiasis in an Outpatient Setting at Amami Oshima Island in Japan: A Case Report

**DOI:** 10.7759/cureus.58851

**Published:** 2024-04-23

**Authors:** Takashi Chinen, Manabu Kameyama, Kensuke Minami

**Affiliations:** 1 Department of Clinical Oncology, Jichi Medical University, Shimotsuke, JPN; 2 Division of General Internal Medicine, Kagoshima Oshima Prefectural Hospital, Amami, JPN; 3 Department of Infection Control, Jichi Medical University, Shimotsuke, JPN

**Keywords:** ivermectin, protein-losing gastroenteropathy, lower extremity edema, hypoalbuminemia, japan, fertilizers, agar, strongyloides stercoralis, strongyloidiasis

## Abstract

Strongyloidiasis is a parasitic infection caused by the nematode *Strongyloides stercoralis* that presents with a variety of nonspecific symptoms. Diagnosis is challenging unless physicians suspect this disease and perform sensitivity tests. We report a case of strongyloidiasis with protein-losing gastroenteropathy-like symptoms in a 92-year-old Japanese female with lower extremity edema and hypoalbuminemia. In this case, the patient refused invasive tests for a complete examination; however, an agar plate culture of a stool sample was used to diagnose strongyloidiasis. The patient was treated with ivermectin during the second visit. One month later, leg edema and hypoproteinemia improved. When the cause of the symptoms is unclear, physicians should be aware of the possibility of strongyloidiasis in a person residing in a tropical or subtropical environment, where human feces are used as fertilizer and individuals frequently go barefoot in agricultural settings.

## Introduction

Strongyloidiasis, a soil-transmitted helminthic disease, is a neglected tropical infection for which the World Health Organization has initiated measures to reduce the number of infected individuals [1]. It is a parasitic infection in humans caused by the nematode *Strongyloides stercoralis*, and its unique asexual autoinfection can lead to persistent infection [2]. Half of the patients with chronic strongyloidiasis are asymptomatic, while the other half present with a variety of nonspecific symptoms, including dermatological, respiratory, and gastrointestinal symptoms. Diagnosis is challenging for physicians in non-endemic areas unless they suspect this disease and perform sensitivity tests [3]. Chronic infection with *S. stercoralis *can be treated with oral ivermectin; however, immunosuppression in a patient can lead to hyperinfection and dissemination of *S. stercoralis*, which can lead to death [4]. As sanitary conditions have improved, people no longer enter fields barefoot, and the use of human waste as fertilizer has been banned in Japan. However, the disease has become more prevalent among older people taking immunosuppressive medications who reside in the subtropical regions of Okinawa and Amami islands, where there is a lack of awareness, resulting in delayed diagnosis and life-threatening disease, even if treatable [5].

This article was previously presented as a meeting poster at the 8th Annual Conference of Japan Primary Care Association on May 13, 2017.

## Case presentation

A 92-year-old Japanese female residing on Amami Oshima Island in Kagoshima Prefecture, Japan, who walked with a cane but was independent in activities of daily living, first visited our outpatient clinic at Setouchi-Cho Hekichi Clinic in 2015. She complained of edema in the lower extremities that did not improve after taking diuretics prescribed by a physician at another hospital, resulting in a 2 kg weight gain in two months. At the initial visit, she had stopped taking medications owing to a lack of efficacy. Her medical history included bile duct jejunostomy for common bile duct lithiasis at 88 years of age and a fracture of the left femoral neck at 89 years of age. A review of the system revealed no changes in food intake, no abdominal pain, and soft stools twice daily.

Physical examination revealed regular heartbeat sounds with no murmur or fast-pitting edema of the lower extremities to the buttocks. Her laboratory values revealed no eosinophilia, normal platelet count, hypoproteinemia, hypoalbuminemia, no proteinuria, normal thyroid function, and normal prothrombin time (Table 1). Echocardiography showed no evidence of heart failure (ejection fraction: 64%, reference range: 61-71%; estimated pulmonary artery systolic pressure: 31 mmHg, reference range: <40 mmHg), and abdominal ultrasonography showed no evidence of liver cirrhosis or inferior vena cava stenosis (8 mm, reference range: <21 mm).

**Table 1:** Initial laboratory results

Laboratory Test	Patient Values	Reference Range
Blood eosinophil count (/μL)	229	70-440
Platelet count (×10^9^/L)	248	130-369
Serum total protein (g/dL)	5.0	6.7-8.3
Serum albumin (g/dL)	2.4	3.8-5.2
Urine protein-to-creatinine ratio (g/gCr)	0.09	<0.15
Thyroid-stimulating hormone (μIU/mL)	2.47	0.61-4.23
Free T4 (ng/dL)	0.96	0.75-1.45
Prothrombin time (%)	91.3	70-130

Because nephrotic syndrome, cirrhosis, hypothyroidism, heart failure, and deep vein thrombosis were excluded as causes of leg edema and hypoalbuminemia, we suspected protein-losing gastroenteropathy, and the patient was referred to another hospital for a complete examination, including upper and lower endoscopy. However, she refused these tests because of the burden of another lower endoscopy, as she had been tested for a benign rectal ulcer four months prior. A stool sample for agar plate culture by an outsourced laboratory obtained during the initial visit to our clinic revealed the presence of *S. stercoralis*, and the patient was diagnosed with strongyloidiasis. At the second visit, she was treated with a single oral dose of 200 μg/kg/day ivermectin, which was repeated after two weeks. At the third visit, one month later, both leg edema and hypoproteinemia had improved. However, she was later found to be infected with human T-cell leukemia virus type 1.

## Discussion

This case of strongyloidiasis with suspected protein-losing gastroenteropathy was promptly diagnosed using an agar plate culture of a stool sample and treated in an outpatient setting in an endemic area of Japan. Management of protein-losing gastroenteropathy is challenging for physicians. This is because the diagnosis of the causative disease is determined by excluding other causes of edema, malabsorption, and hypoalbuminemia and requires many tests; even when the causative disease is diagnosed, it often does not improve without treatment, and long-term symptom management is necessary [6]. Strongyloidiasis is endemic to Amami Oshima Island, and we encountered another case in which protein-leaking gastroenteropathy-like symptoms were found to be symptoms of strongyloidiasis on examination with invasive testing; in fact, several similar cases have been reported [7,8]. If strongyloidiasis is suspected, a diagnosis can be made, and the treatment is simple. However, if the disease is not suspected, diagnosis is delayed, leading to severe illness and death. In this case, previous physicians may not have considered strongyloidiasis in the differential diagnosis of hypoalbuminemia in older patients with high morbidity in an endemic area of Japan.

The problem with making a diagnosis is that, even if strongyloidiasis is suspected, it can be missed with stool microscopy, the standard method of examination for parasites, because of its low sensitivity of approximately 10% [3]. Since agar plate culture has a high sensitivity of 60-98%, repeat agar plate cultures should be performed first if the disease is suspected [3,9,10]. Serological tests with the risk of false positives due to cross-reactivity and false negatives due to immunosuppression, as well as molecular tests, are not covered by insurance in Japan; therefore, it is difficult to perform these tests primarily when strongyloidiasis is suspected. In this case, the patient had symptoms of a benign rectal ulcer four months prior, and the course of the disease seemed to be one of the symptoms of strongyloidiasis; however, despite a biopsy of the ulcer site, a diagnosis of strongyloidiasis was not made.

Strongyloidiasis may be not a disease in older Japanese people. A 2017 study estimated that the incidence of strongyloidiasis was 8.1% worldwide, particularly in Southeast Asia with the highest incidence of 12.1% [11]. The number of foreign immigrants coming to Japan as part of the labor force is increasing [12], with the majority coming from Southeast Asia, followed by Vietnam, China, and the Philippines [13]. Surveys conducted in 2016 and 2017 in Northern Vietnam showed that the seroprevalence of strongyloidiasis was approximately 20% in all generations [14]. Although strongyloidiasis has become a disease of the past in some older Japanese individuals, some chronically infected immigrants may develop strongyloidiasis in the future. In addition, although Japan’s sewerage population penetration rate is 80.6%, there are still places where sewage treatment is inadequate, particularly on remote islands and depopulated mountainous areas [15,16]. If there is a habit of using human feces as fertilizer or entering fields barefoot, it is theoretically possible that new strongyloidiasis infections occur because of the existence of *S. stercoralis* infection routes. The parasitic infection can be prevented by not using human feces as fertilizer or by not entering the fields barefoot.

## Conclusions

The patient in this study presented with lower extremity edema and hypoalbuminemia and was promptly diagnosed with strongyloidiasis using agar plate cultures of stool samples. When the cause of symptoms or findings is unclear, physicians should be aware of the possibility of strongyloidiasis in a person residing in a tropical or subtropical environment, where human feces are used as fertilizers and individuals frequently go barefoot in agricultural settings. In Japan, strongyloidiasis can be diagnosed simply and reliably using agar plate cultures of stool samples.
